# The prevalence and risk factors study of cognitive impairment: Analysis of the elderly population of Han nationality in Hunan province, China

**DOI:** 10.1111/cns.14478

**Published:** 2023-09-22

**Authors:** Tianyan Xu, Guiwen Bu, Li Yuan, Lu Zhou, Qijie Yang, Yuan Zhu, Sizhe Zhang, Qianqian Liu, Ziyu Ouyang, Xuan Yang, Beisha Tang, Bin Jiao, Yuzhang Bei, Lu Shen

**Affiliations:** ^1^ Department of Neurology, Xiangya Hospital Central South University Changsha China; ^2^ Department of Neurology Liuyang Jili Hospital Changsha China; ^3^ National Clinical Research Center for Geriatric Disorders Central South University Changsha China; ^4^ Engineering Research Center of Hunan Province in Cognitive Impairment Disorders Central South University Changsha China; ^5^ Hunan International Scientific and Technological Cooperation Base of Neurodegenerative and Neurogenetic Diseases Changsha China; ^6^ Key Laboratory of Hunan Province in Neurodegenerative Disorders Central South University Changsha China; ^7^ Key Laboratory of Organ Injury Aging and Regenerative Medicine of Hunan Province Changsha China

## Abstract

**Objective:**

A large number of studies have found that the prevalence of cognitive impairment varies in different regions. However, data on cognitive impairment in the Chinese population is still lacking. The goal of this study was to assess the prevalence of cognitive impairment among the elderly in a region of China and explore the associated risk factors.

**Methods:**

We performed a population‐based cross‐sectional survey from April to June 2022. Residents come from three villages and six urban communities in the county‐level city of Liuyang in southern China (*N* = 3233) and the coverage rate of our study population reached 73%. Participants were assessed with a series of clinical examinations and neuropsychological measures. A total of 2598 participants were selected after filtering out those under 60 years old or with incomplete data. Patients with cognitive impairment included those with mild cognitive impairment (MCI) or dementia who met standard diagnostic criteria.

**Results:**

The prevalence of cognitive impairment, MCI, and dementia among participants aged 60 years and older were 21.48% (95% CI, 19.90–23.10), 15.70% (95% CI, 14.30–17.10), and 5.77 (95% CI, 4.90–6.70), respectively. And residents in villagers were more likely to have cognitive impairment than in urban communities (*p* < 0.001). Age growth and education level were independent influencing factors for cognitive impairment in all populations (*p* < 0.001). For lifestyles factors, both smoking and drinking reduced the risk of cognitive impairment (*p* < 0.05), but when further quantified, the link disappeared. Moreover, having cerebrovascular disease and severe vision impairment were risk factors (*p* < 0.05).

**Conclusion:**

A representative prevalence of cognitive impairment, MCI, and dementia was found in the elderly Han Chinese population in Southern China. And we further explored the role of known risk factors, particularly in physical activity, smoking, and alcohol consumption.

## Graphical abstract

We performed a cross‐sectional study on the cognitive function of the Han elderly population in southern China and the target population coverage rate was high, reaching 73%. A representative prevalence rate of cognitive impairment was obtained, and the relevant risk factors were comprehensively analyzed.

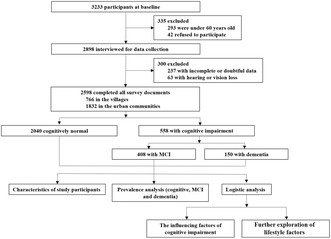

## 1 INTRODUCTION

Cognitive impairment, such as mild cognitive impairment (MCI) and dementia, is increasingly damaging the health of people around the world, and dementia is emerging as the leading cause of disability in people older than 65 years worldwide, including in China.^1^, ^2^ Nowadays, there are 40–50 million people living with dementia, and the number of dementia patients in China accounts for approximately 25% of the global total.^3^ There are many studies have focused on the prevalence of dementia in China, and the results showed a broad range, from 5.0% to 7.7% for individuals aged 60 years and older and from 2.0% to 13.0% for individuals aged 65 years and older.^4^ The differences in the prevalence might be explained by different dementia survival times, environmental risk factors and genetic factors, and mortality before the onset of dementia.^4^

Many factors can influence the prevalence of cognitive impairment. Due to China's large size and wide longitude, it varies from region to region. Jia and colleagues found that the prevalence of dementia and Alzheimer's disease (AD) is significantly higher in rural areas than in urban areas, and education may be an important reason for the urban–rural difference.^5^ Another study found that the number of dementia patients varied in different geographical regions, with northern China (5.5%) being higher than southern China (4.8%).^6^ Gender differences also play a role. Rudan and colleagues discovered that the gender difference in the prevalence of AD was also higher (the ratio of women to men was 2.37).^7^ And The Global Burden of Diseases, Injuries, and Risk Factors (GBD) Study 2016 found that four risk factors were judged to have sufficient evidence for a causal link to AD and other dementias: high BMI, high fasting plasma glucose, smoking, and high intake of sugar‐sweetened beverages.^3^ For example, passive smoking exposure increased the risk of cognitive impairment in older adults, especially non‐smokers.^8^ Co‐existence of smoking and regular alcohol drinking at midlife increased the risk of cognitive impairment, had a much stronger impact than the individual factors on risk of cognitive impairment in late life.^9^ But there is still controversy about drinking. Observational studies have suggested that light‐to‐moderate alcohol consumption decreases the risk of Alzheimer's disease.^10^, ^11^ However, other study has found no evidence of a causal relationship between alcohol consumption or alcohol dependence and late‐onset Alzheimer's disease (LOAD).^12^ Because the influence of various factors on cognitive impairment is not clear, the influence of them, such as demographic characteristics, lifestyle, and comorbidities, on the incidence of cognitive impairment is worth exploring.

At present, there are still few comprehensive risk factor surveys on large‐scale cognitive impairment in China. Our study focused on all elderly people in different communities in Liuyang City, Hunan Province, with a high survey coverage, to explore new findings on risk factors for cognitive impairment.

## 2 METHODS

### 2.1 Study design and participants

**FIGURE 1:**
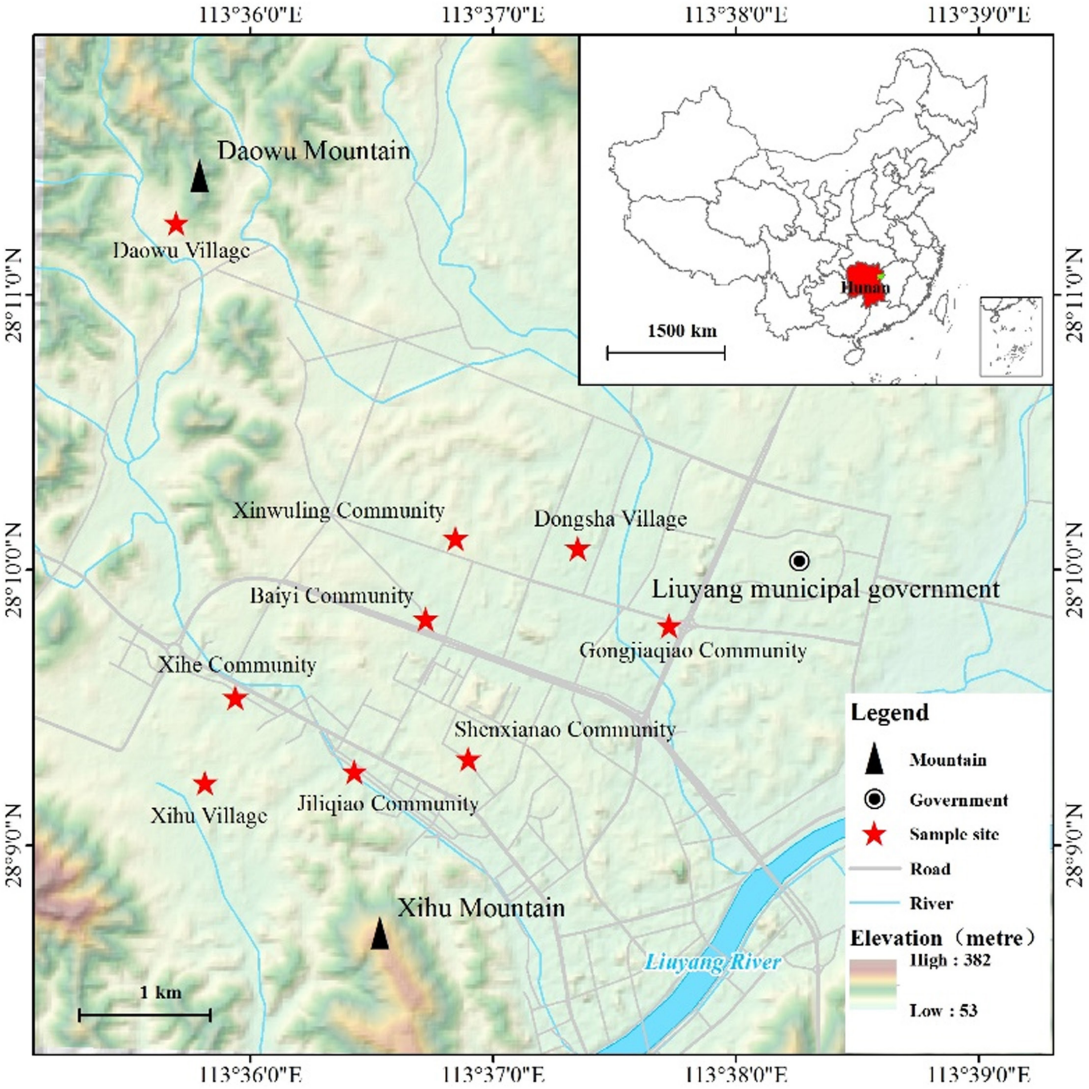
Geographical distribution of study locations. Nine communities (Xihu Village, Daowu Village, Dongsha Village, Gongjiaqiao Community, Xinwuling Community, Xihe Community, Baiyi Community, Shenxianao Community, and Jiliqiao Community) in the Jili subdistrict were selected as the research targets, and their positions in the subdistrict were marked on the map.

**FIGURE 2:**
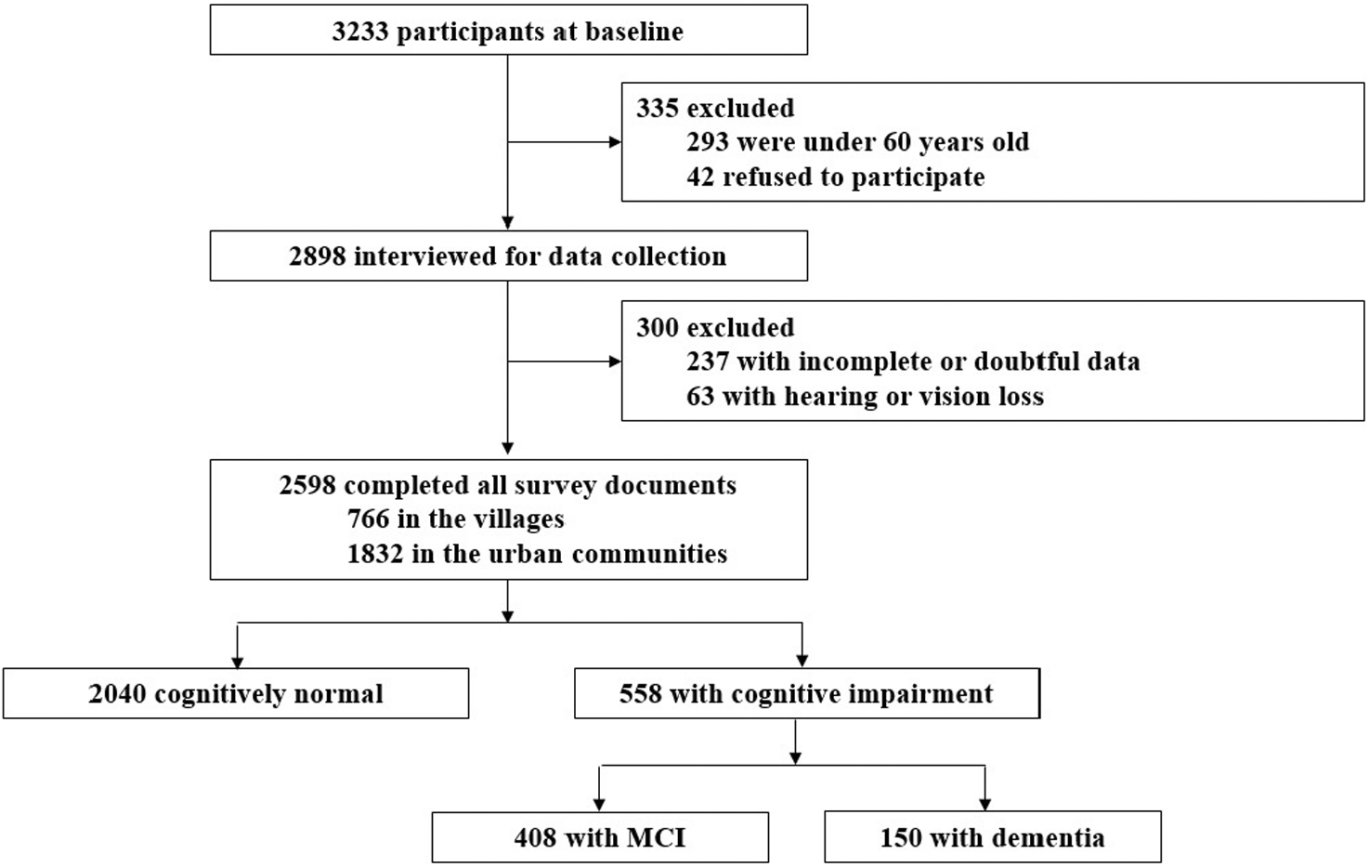
Study flow chart.

This study is a population‐based cross‐sectional survey conducted from April to June 2022 among individuals from Jili Subdistrict, Liuyang county‐level City, Changsha City, Hunan Province, China. Jili Subdistrict consists of three villages (Xihu Village, Daowu Village, and Dongsha Village) and six urban communities (Gongjiaqiao Community, Xinwuling Community, Xihe Community, Baiyi Community, Shenxianao Community, and Jiliqiao Community) (Figure 1). Villages are remote and far away from the downtown, while urban communities are closer to the downtown, with convenient transportation and modern life. Using a cluster sampling design, we recruited separate participants (*N* = 3233) from each of the nine communities. Participants were screened and the removal criteria were: (1) age <60 years old; (2) refusal to participate; (3) incomplete or doubtful data; (4) Severe hearing or vision loss that prevents completion of cognitive assessments. After filtering out those who did not meet the criteria, 2598 samples remained (Figure 2). According to the elderly resident population data collected by the local government, the coverage rate of our study population reached 73%.

### 2.2 Demographic characteristics of study participants

We screened the demographic characteristics of participants through the questionnaire survey, including age, gender, education level, location, waistline, body mass index (BMI), blood lipid, lifestyle, and personal medical history. We collected fasting blood samples between 7 and 9 a.m. and tested fasting blood glucose and blood lipids at Jili Hospital. The blood lipid indexes tested included: triglycerides (TG), total cholesterol (TC), low‐density lipoprotein cholesterol (LDL‐C), and high‐density lipoprotein cholesterol (HDL‐C). In addition, we performed electrocardiography (ECG) and vision examination.

Educational attainment was classified as illiteracy (0 years), primary school (1–6 years), and secondary school or higher (>6 years). Body mass index (BMI) was calculated as the weight (kg) divided by the square of the height (m^2^). Lifestyle factors included physical exercise (exercising for more than 30 min a week for more than half a year; including the frequency of physical activity, time of day, and years of physical exercise), cigarette smoking (regular smoking for at least 6 months before the survey; including the number of cigarettes smoked per day and years of smoking), and alcohol consumption (regular drinking for at least 1 year before the survey, including the frequency of drinking, daily consumption, and years of drinking). According to the previous medical records and the examination results collected this time, we determined whether the patients had hypertension, diabetes, cerebrovascular disease, heart disease, and severe visual impairment.

### 2.3 Assessment and diagnosis of cognitive function

The neuropsychological assessment team is composed entirely of neurology experts and neurology doctoral students, all of whom are proficient in neuropsychological assessment scales. We used the mini‐mental state examination (MMSE), with a score ranging from 0 to 30, to assess cognitive function.^1^ Since MMSE scores are easily influenced by educational level, we set the cut‐off scores for defining cognitive impairment of different educational backgrounds as follows: illiterate participants ≤17 points; primary‐education participants, ≤20 points; and secondary‐ or higher‐education participants, ≤24 points. Then we administered the clinical dementia rating (CDR) scale, which includes memory, orientation, judgment and problem‐solving, community affairs, home and hobbies, and personal care, to obtain information on cognitive complaints and activities of daily living.^2^ All scores collected were reviewed by the expert pane, and diagnoses were made at the end of each workday.

Based on MMSE and CDR scores, participants were classified into two categories: normal cognitive functioning and cognitive impairment, with the latter further classified as MCI and dementia. The MCI diagnostic criteria are listed below^3^, ^4^: (1) one or more domains in CDR scored ≥0.5 points, global CDR score ≤0.5 points; (2) essentially preserved daily and social functioning; (3) no dementia. Diagnostic criteria for dementia were based on the fourth edition of the Diagnostic and Statistical Manual of Mental Disorders.^5^

### 2.4 Statistical analysis

We compared the characteristics according to the presence of cognitive impairment, using chi‐square tests for categorical variables, unpaired *t*‐test for interval ones, and Mann–Whitney *U*‐test for variables that are not normally distributed. Moreover, estimates of the prevalence of cognitive impairment, MCI, and dementia were calculated separately for the overall population and subgroups stratified by age and gender. Age‐standardized prevalence rates were calculated based on the official population data files of the local government. Logistic regression models were used to ascertain the risk factors associated with cognitive impairment considering the main effects of sociodemographic characteristics, BMI, blood lipid, lifestyle, comorbidity, and medication, and to explore whether there were differences in influencing factors between villages and urban communities. We used IBM SPSS Statistics 23.0 for statistical analyses. Differences between the groups were considered statistically significant when the *p* value was less than 0.05.

## 3 RESULTS

### 3.1 Characteristics of study participants

A total of 2598 participants completed the survey, and the characteristics of all samples are presented in Table 1. The median age of all participants was 69.00 (IQR 66.00–73.00) years and 54.0% were women. The median years of education were 6.00 (IQR 3.00–9.00) years, and there were more participants in urban communities than in villages. Cognitive impairment was present in 558 (21.48%) participants, and people were older, more female, and had fewer years of formal education (*p* < 0.05). Additionally, the median waistline and BMI were 79.00 (IQR 73.15–85.00) cm and 24.36 (IQR 22.28–26.35) kg/m^2^, with no significant difference between cognitive impairment and cognitively normal populations. Blood lipid indicators showed the same results. In terms of lifestyle, cognitive impairment individuals were less likely to do physical exercise, smoke, and drink than cognitively normal individuals (*p* < 0.05). According to comorbid conditions, the proportions of individuals with hypertension and severe vision impairment were higher in cognitive impairment individuals (*p* < 0.05).

**TABLE 1:** Characteristics of participants according to the presence of cognitive impairment (*n* = 2598).

Characteristics	All (*N* = 2598)	Cognitive impairment		*p*
		No (*N* = 2040)	Yes (*N* = 558)	
Age (years), median (IQR)	69.00 (66.00–73.00)	68.00 (66.00–72.00)	70.00 (66.00–75.00)	<0.001^b^
Gender, Female, *N* (%)	1404 (54.0)	1076 (52.7)	328 (58.8)	0.011^a^
Education (years), median (IQR)	6.00 (3.00–9.00)	6.00 (3.00–9.00)	4.00 (1.50–9.00)	<0.001^b^
Region, *N* (%)				
Villages	766 (29.5)	540 (70.5)	226 (29.5)	<0.001^a^
Urban communities	1832 (70.5)	1500 (81.9)	332 (18.1)	<0.001^a^
Waistline (cm), median (IQR)	79.00 (73.15–85.00)	79.00 (74.00–85.00)	78.30 (72.75–85.00)	>0.05^b^
BMI (kg/m^2^), median (IQR)	24.36 (22.28–26.35)	24.35 (22.30–26.35)	24.27 (22.11–26.39)	>0.05^b^
Blood lipid				
TC (mmol/L), median (IQR)	4.97 (4.31–5.70)	4.93 (4.29–5.69)	5.00 (4.34–5.77)	>0.05^b^
TG (mmol/L), median (IQR)	1.29 (0.91–1.86)	1.26 (0.91–1.89)	1.27 (0.87–1.87)	>0.05^b^
LDL‐C (mmol/L), median (IQR)	3.09 (2.47–3.64)	3.04 (2.45–3.65)	3.09 (2.50–3.71)	>0.05^b^
HDL‐C (mmol/L), median (IQR)	1.46 (1.25–1.66)	1.44 (1.23–1.66)	1.47 (1.26–1.70)	>0.05^b^
Physical exercise, *N* (%)	1840 (70.8)	1483 (72.7)	357 (64.0)	<0.001^a^
Cigarette smoking, *N* (%)	678 (26.1)	557 (27.3)	121 (21.7)	0.007^a^
Alcohol consumption, *N* (%)	280 (10.8)	246 (12.1)	34 (6.1)	<0.001^a^
Comorbidity, *N* (%)				
Hypertension	1501 (57.8)	1152 (56.5)	349(62.5)	0.010^a^
Diabetes	345 (13.3)	261 (12.8)	84 (15.1)	>0.05^a^
Cerebrovascular disease	93 (3.6)	67 (3.3)	26 (4.7)	>0.05^a^
Cardiovascular disease	113 (4.3)	88 (4.3)	25 (4.5)	>0.05^a^
Severe vision impairment	391 (15.1)	257 (12.6)	134 (24.0)	<0.001^a^

Abbreviations: HDL‐C, high‐density lipoprotein cholesterol; LDL‐C, low‐density lipoprotein cholesterol; TC, total cholesterol; TG, triglyceride.

^a^ Chi‐square test.

^b^ Mann–Whitney *U* test.

### 3.2 Prevalence of cognitive impairment

As shown in Table 2, the prevalence of cognitive impairment, MCI, and dementia among participants aged 60 years and older was 21.48% (95% CI, 19.90–23.10), 15.70% (95% CI, 14.30–17.10), and 5.77 (95% CI, 4.90–6.70), respectively. For village dwellers, the rate were 29.50% (95% CI, 26.30–32.70) for cognitive impairment, 17.36% (95% CI, 14.70–20.10) for MCI, and 6.27% (95% CI, 4.50–8.00) for dementia. The corresponding figures for the urban communities population were 18.12% (95% CI, 16.40–19.90), 15.01% (95% CI, 13.40–16.60), and 5.57% (95% CI, 4.50–6.60). To determine the difference between villages and urban communities, we standardized our data according to age using local government demographic data. The rate of cognitive impairment was significantly higher in villages than in urban communities (*p* < 0.05).

**TABLE 2:** Prevalence of cognitive impairment, MCI, and dementia, in all, village, and urban communities population.

Prevalence	All communities^a^	Villages^a^	Urban communities^a^	*p* ^b^
Cognitive impairment				
Crude	21.48 (19.90–23.10)	29.50 (26.30–32.70)	18.12 (16.40–19.90)	<0.05
Standardized	21.40 (21.20–21.60)	28.20 (28.00–28.40)	18.45 (18.30–18.60)	
MCI				
Crude	15.70 (14.30–17.10)	17.36 (14.70–20.10)	15.01 (13.40–16.60)	>0.05
Standardized	16.12 (16.00–16.30)	17.61 (17.50–17.80)	15.48 (15.30–15.60)	
Dementia				
Crude	5.77 (4.90–6.70)	6.27 (4.50–8.00)	5.57 (4.50–6.60)	>0.05
Standardized	5.80 (5.70–5.90)	5.83 (0.057–0.059)	5.69 (5.60–5.80)	

Abbreviation: MCI, mild cognitive impairment.

^a^ Prevalence (percent) and 95% confidence intervals (in parentheses) are provided.

^b^ Chi‐square test.

**FIGURE 3:**
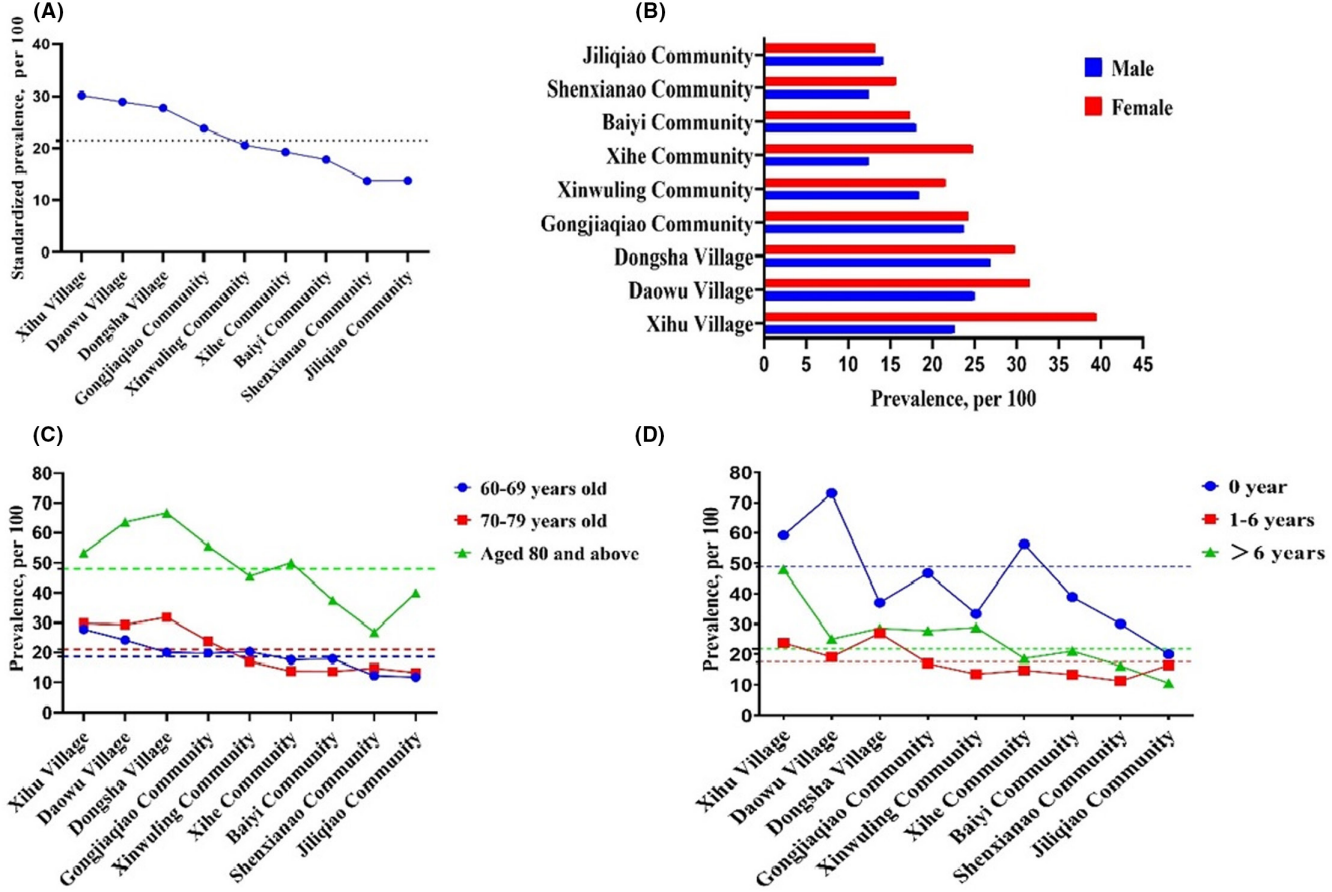
Prevalence of cognitive impairment. (A) Prevalence rates among different communities; (B) prevalence rates by gender category among different communities; (C) prevalence rates by age category among different communities. (D) Prevalence rates by education level category among different communities. The dashed line shows the average level for each group.

Additionally, the prevalence rates for subgroups stratified by gender, age, and education level are presented in Figure 3. In most communities, the prevalence of cognitive impairment was higher in women than in men, except in Baiyi Community and Jiliqiao community. Stratified by age, the prevalence rates generally increased with age, and the prevalence rate of people aged 80 years and above was markedly higher than that of other age groups, with the highest prevalence reaching 66.7% (Dongsha Village). But the pattern varied in some communities. The prevalence rates of people aged 70–79 in Xinwulin, Xihe, and Baiyi communities were lower than in other communities. In terms of the education level, we found mostly the illiterate group had a much higher prevalence rate than the other two groups. Notably, years of education were not completely inversely associated with the prevalence. Those with more than 6 years of education had a higher prevalence than those with 1–6 years of education in most communities.

**FIGURE 4:**
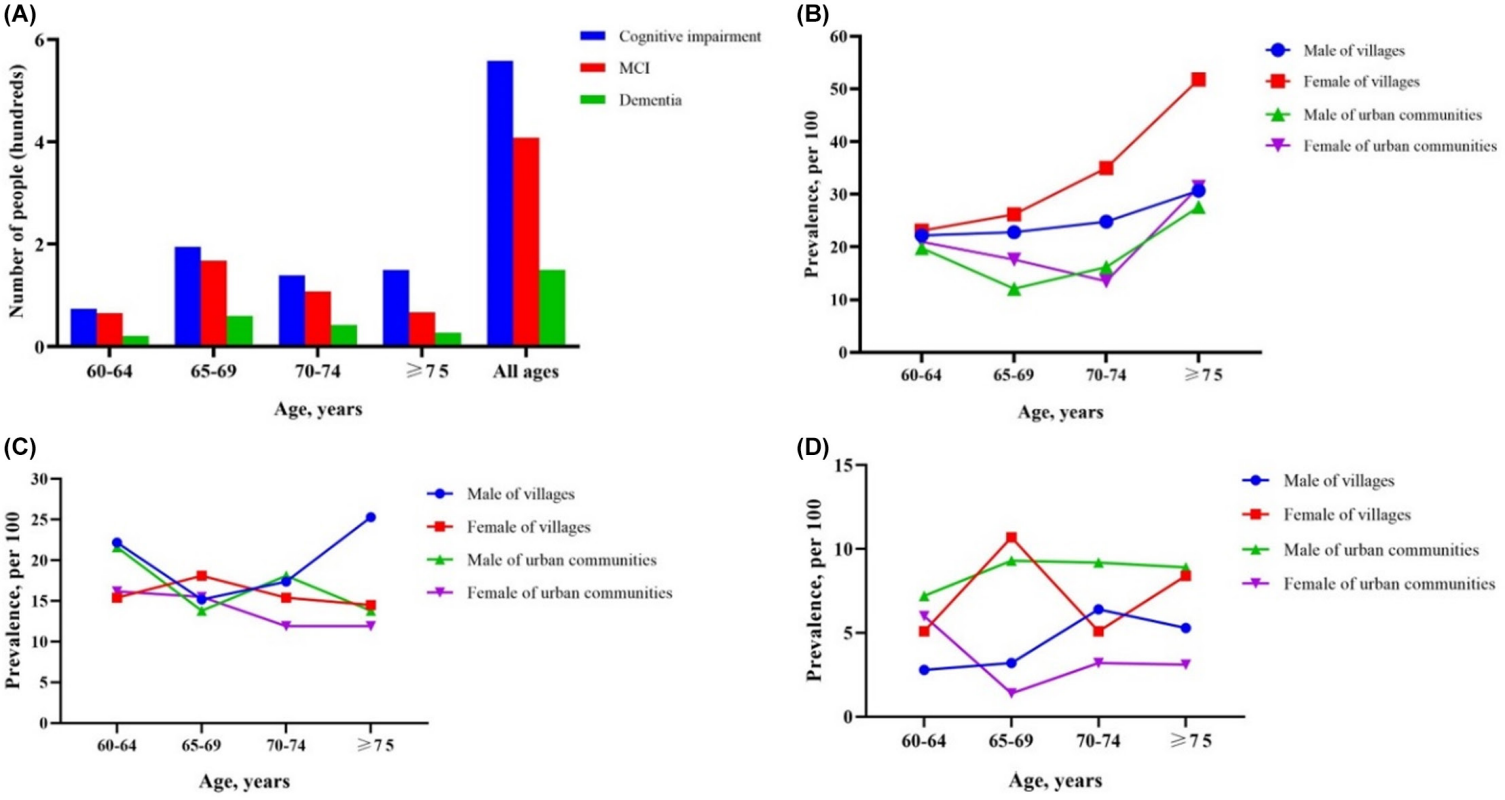
Prevalence of cognitive impairment, MCI, and dementia for villages and urban communities stratified by gender and age. (A) Prevalence rates in total individuals; (B) prevalence rates for cognitive impairment; (C) prevalence rates for MCI. (D) Prevalence rates for dementia. MCI, mild cognitive impairment.

To further compare the differences in the prevalence rate of residents in villages and urban communities, we stratified the two populations by age and gender (Figure 4). Across all age groups, MCI patients outnumbered those with dementia. In both villages and urban communities population, the prevalence of cognitive impairment was higher in females than in males, and it gradually increased with age, up to 51.8%. But there is a downward trend in the prevalence of the urban community population between the ages of 60 and 69. This overall trend was also seen in dementia, but the rate for males in urban communities was higher than in the other three groups. In terms of MCI, the increasing prevalence with age was not obvious, and there is little difference in all age groups. It is worth noting that among the male village population, the prevalence is higher in both the 60–64 and over 75 age groups.

### 3.3 Logistic analysis

#### 3.3.1 The influencing factors of cognitive impairment

To identify possible risk factors, we performed multivariate logistic regression analysis on the samples of patients (cognitive impairment) and cognitively normal individuals (Table 3). We found that people have a 4.6% significantly higher likelihood to have cognitive impairment for each additional year of age (odds ratio [OR], 1.046; 95% CI, 1.027–1.065), and educated people have a lower risk of cognitive impairment compared to illiterate people (primary school: odds ratio [OR], 0.273, 95% CI, 0.199–0.374; Secondary school or higher: odds ratio [OR], 0.504, 95% CI, 0.355–0.716). Among the indicators of lifestyle, alcohol consumption was a protective factor for having a cognitive impairment (odds ratio [OR], 0.502; 95% CI, 0.330–0.764), while physical exercise and cigarette smoking were not significantly associated. The risk for cognitive impairment was greater among individuals with cerebrovascular disease (odds ratio [OR], 2.477; 95% CI, 1.371–4.475) and severe vision impairment (odds ratio [OR], 1.686; 95% CI, 1.328–2.140), and there were no significant differences in individuals with hypertension, diabetes, and heart disease. As for gender, BMI, and lipid levels, we did not have any significant findings.

**TABLE 3:** Logistic regression models for cognitive impairment in our populations^a^.

Index	All participants		Villages		Urban communities	
	OR (95% CI)	*p*	OR (95% CI)	*p*	OR (95% CI)	*p*
Age	1.046 (1.027–1.065)	<0.001	1.072 (1.039–1.106)	<0.001	1.032(1.010–1.056)	0.005
Education level (years)						
0	Ref.		Ref.		Ref.	
1–6	0.273 (0.199–0.374)	<0.001	0.226 (0.142–0.361)	<0.001	0.291(0.188–0.451)	<0.001
>6 years	0.504 (0.355–0.716	<0.001	0.447 (0.238–0.840)	0.012	0.505(0.322–0.791)	0.003
Gender						
Male	Ref.		Ref.		Ref.	
Female	0.999 (0.769–1.298)	0.995	1.059 (0.673–1.665)	0.805	0.965(0.698–1.334)	0.830
BMI	0.997 (0.967–1.029)	0.872	1.000 (0.948–1.054)	0.995	1.003(0.962–1.046)	0.887
Blood lipid						
TG	0.886 (0.677–1.161)	0.381	0.682 (0.435–1.071)	0.097	1.186(0.869–1.618)	0.282
TC	1.081 (0.992–1.179)	0.075	1.039 (0.896–1.205)	0.610	1.072(0.968–1.188)	0.184
LDL‐C	1.072 (0.816–1.409)	0.616	1.396 (0.918–2.121)	0.119	0.786(0.567–1.090)	0.150
HDL‐C	1.481 (0.951–2.306)	0.082	1.839 (0.900–3.759)	0.095	1.170(0.672–2.038)	0.578
Physical exercise						
No	Ref.		Ref.		Ref.	
Yes	0.907 (0.718–1.146)	0.415	0.989 (0.699–1.399)	0.950	0.815(0.597–1.113)	0.198
Cigarette smoking						
No	Ref.		Ref.		Ref.	
Yes	0.891 (0.660–1.202)	0.449	0.825 (0.502–1.355)	0.447	0.956(0.654–1.396)	0.814
Alcohol consumption						
No	Ref.		Ref.		Ref.	
Yes	0.502 (0.330–0.764)	0.001	0.590 (0.311–1.119)	0.106	0.432(0.243–0.767)	0.004
Hypertension						
No	Ref.		Ref.		Ref.	
Yes	1.176 (0.956–1.446)	0.125	1.091 (0.764–1.557)	0.632	1.198(0.927–1.548)	0.167
Diabetes						
No	Ref.		Ref.		Ref.	
Yes	1.255 (0.927–1.700)	0.141	1.113 (0.615–2.014)	0.723	1.213(0.852–1.728)	0.283
Cerebrovascular disease						
No	Ref.		Ref.		Ref.	
Yes	2.477 (1.371–4.475)	0.003	5.133 (1.831–14.393)	0.002	1.666(0.774–3.585)	0.192
Cardiovascular disease						
No	Ref.		Ref.		Ref.	
Yes	0.871 (0.494–1.538)	0.635	0.943 (0.405–2.197)	0.892	0.864(0.401–1.862)	0.708
Severe vision impairment						
No	Ref.		Ref.		Ref.	
Yes	1.686 (1.328–2.140)	<0.001	1.656 (1.118–2.452)	0.012	1.689(1.249–2.284)	0.001

Abbreviations: HDL‐C, high‐density lipoprotein cholesterol; LDL‐C, low‐density lipoprotein cholesterol; TC, total cholesterol; TG, triglyceride.

^a^ The covariates included age, education levels, gender, community, BMI, TC, TG, HDL‐C, LDL‐C, physical exercise, cigarette smoking, alcohol consumption, and comorbidity.

We also compared people in different communities. Age (village: odds ratio [OR], 1.072; 95% CI, 1.039–1.106; urban community: odds ratio [OR], 1.032, 95% CI, 1.010–1.056) and education level (village: primary school, odds ratio [OR], 0.226; 95% CI, 0.142–0.361, secondary school or higher, odds ratio [OR], 0.447; 95% CI, 0.238–0.840; urban community: primary school, odds ratio [OR], 0.291, 95% CI, 0.188–0.451, secondary school or higher, odds ratio [OR], 0.505; 95% CI, 0.322–0.791) remained significant factors for cognitive impairment in both villages and urban communities. Interestingly, alcohol consumption was protective only in urban community populations (odds ratio [OR], 0.432; 95% CI, 0.243–0.767). Furthermore, we found that village residents have a 13.3% significantly higher likelihood to have cognitive impairment for having a cerebrovascular disease (odds ratio [OR], 5.133; 95% CI, 1.831–14.393). For people with severe vision impairment, it increased risk of cognitive impairment regardless of where they live (villages, odds ratio [OR], 1.656; 95% CI, 1.118–2.452; urban community, odds ratio [OR], 1.689; 95% CI, 1.249–2.284).

#### 3.3.2 Age and education levels are independent influencing factors for cognitive impairment

During the epidemiological investigation, we found differences in the age span and education levels of participants between communities, with some communities having a younger population and some having a more educated population. So we further analyzed the influence of these two factors (Table 4). It revealed that the education level was a protective factor (Primary school: odds ratio [OR], 0.242 95% CI, 0.179–0.327; Secondary school or higher: odds ratio [OR], 0.344, 95% CI, 0.250–0.473), whereas that age (odds ratio [OR], 1.056; 95% CI, 1.038–1.074) was a risk factor when unadjusted for covariates (Model 1). Then we adjusted for gender and region (Model 2) and the impact trend did not change (age: odds ratio [OR], 1.057; 95% CI, 1.039–1.075; Primary school: odds ratio [OR], 0.277, 95% CI, 0.203–0.377; Secondary school or higher: odds ratio [OR], 0.506, 95% CI, 0.359–0.714). Additionally, we added the rest covariates to the regression models (Model 3) to test their significance. After controlling the all confounding factors, we discovered that individuals who are older or less educated are more likely to have cognitive impairment (age: odds ratio [OR], 1.046 95% CI, 1.027–1.065; Primary school: odds ratio [OR], 0.273, 95% CI, 0.199–0.374; Secondary school or higher: odds ratio [OR], 0.504, 95% CI, 0.355–0.716).

**TABLE 4:** Logistic regression analysis of age and education level.

Index	Model 1^a^		Model 2^b^		Model 3^c^	
	OR (95% CI)	*p*	OR (95% CI)	*p*	OR (95% CI)	*p*
Age	1.056 (1.038–1.074)	<0.001	1.057 (1.039–1.075)	<0.001	1.046 (1.027–1.065)	<0.001
Education level						
Illiteracy (0 years)	Ref.		Ref.		Ref.	
Primary school (1–6 years)	0.242 (0.179–0.327)	<0.001	0.277 (0.203–0.377)	<0.001	0.273 (0.199–0.374)	<0.001
Secondary school or higher (>6 years)	0.344 (0.250–0.473)	<0.001	0.506 (0.359–0.714)	<0.001	0.504 (0.355–0.716)	<0.001

^a^ Model 1 was unadjusted.

^b^ Model 2 was adjusted for gender and region.

^c^ Model 3 was adjusted for all covariates, including age, education levels, gender, community, BMI, TC, TG, HDL‐C, LDL‐C, physical exercise, cigarette smoking, alcohol consumption, and comorbidity.

Consequently, after the adjustment of all covariates, the association weakened but was still statistically significant, indicating that age and education levels are independent influencing factors for cognitive impairment.

#### 3.3.3 Further exploration of lifestyle factors

To further explore the role of lifestyle factors in cognitive dysfunction, we further collected more detailed data, including the frequency of physical exercise, daily physical exercise time, years of physical exercise, daily smoking number, years of smoking, frequency of drinking, daily alcohol consumption (grams of pure ethanol), and years of drinking (Table 5). The result was not quite the same as before. We only found that an increase in daily smoking number was a protective factor for cognitive impairment (odds ratio [OR], 0.969; 95% CI, 0.944–0.994). In addition, a higher frequency of physical activity and a longer duration of exercise were associated with a lower risk of cognitive impairment among all individuals. But we did not find a trend in alcohol‐related consent.

**TABLE 5:** Logistic regression models for the lifestyle factors^a^.

Index	All participants		Villages		Urban communities	
	OR (95% CI)	*p*	OR (95% CI)	*p*	OR (95% CI)	*p*
Frequency of physical exercise						
Never	Ref.		Ref.		Ref.	
1–2 times a week	1.117 (0.680–1.833)	0.662	1.610 (0.520–4.981)	0.409	1.175 (0.656–2.103)	0.588
3–6 times a week	0.707 (0.395–1.265)	0.243	0.612 (0.086–4.360)	0.624	0.877 (0.457–1.681)	0.692
At least 7 times a week	0.915 (0.470–1.782)	0.794	0.639 (0.0181–2.261)	0.487	1.133 (0.474–2.710)	0.779
Daily physical exercise time (minutes)	0.987 (0.973–1.000)	0.055	0.992 (0.959–1.026)	0.648	0.988 (0.973–1.003)	0.107
Years of physical exercise (years)	1.002 (0.974–1.031)	0.900	0.953 (0.889–1.022)	0.174	1.011 (0.978–1.044)	0.523
Daily smoking number (counts)	0.969 (0.944–0.994)	0.014	0.964 (0.928–1.002)	0.060	0.971 (0.942–1.001)	0.055
Years of smoking (years)	1.031 (0.990–1.036)	0.268	1.007 (0.992–1.021)	0.377	1.009 (0.997–1.021)	0.150
Frequency of drinking						
Never	Ref.		Ref.		Ref.	
1–2 times a week	0.411 (0.115–1.477)	0.173	0.409 (0.054–3.118)	0.389	0.580 (0.102–3.291)	0.539
3–6 times a week	0.889 (0.231–3.414)	0.864	0.728 (0.078–6.853)	0.781	1.154 (0.191–6.952)	0.876
At least 7 times a week	0.636 (0.183–2.206)	0.476	0.289 (0.038–2.193)	0.230	1.220 (0.240–6.201)	0.810
Daily alcohol consumption (grams of pure ethanol)						
<20 g	Ref.		Ref.		Ref.	
20–60 g	1.014 (0.145–2.478)	0.975	0.889 (0.230–3.431)	0.865	1.255 (0.367–4.288)	0.717
>60 g	1.095 (0.302–3.969)	0.890	2.517 (0.395–16.043)	0.329	0.392 (0.038–4.005)	0.429
Years of drinking (years)	1.011 (0.986–1.036)	0.388	1.015 (0.976–1.055)	0.459	1.002 (0.970–1.036)	0.885

^a^ All data were adjusted for gender, age, and education level.

## 4 DISCUSSION

Over the past decades, many studies have focused on dementia in China, with large numbers of studies focusing on comparing the prevalence between different cities or between cities and villages or exploring differences between China and foreign countries. Our study is a population‐based cross‐sectional investigation conducted among Chinese elderly individuals, with participation covering 73% of the target population. Some new results were found through a comprehensive data collection of participants.

Our data showed that the prevalence rates of cognitive impairment, MCI, and dementia among Chinese elderly individuals aged 60 years and older were 21.48%, 15.70%, and 5.77%, respectively. According to the large and authoritative multi‐center large sample epidemiological survey of cognitive impairment, the prevalence of MCI and dementia are about 15.5% and 6.00% in Chinese elderly people,^13^ which is almost consistent with our corresponding data. In addition, the prevalence rates of people in different living environments were different, showing that village residents are significantly higher than urban community residents. And prevalence rates in village and urban communities are also generally consistent with known data.^5^, ^14^

The prevalence stratified by gender, age, and education level in our study showed that it was higher in women than in men and gradually increased with aging and lack of education. After stratifying the population by gender and age, the prevalence rate of cognitive impairment, MCI, and dementia showed a trend of increasing with age, and it was generally higher in women than in men and was most obvious in cognitive impairment. And we further demonstrated that age growth and education level were independent influencing factors for cognitive impairment in all populations. Gender, age, and education are the most studied risk factors associated with cognitive impairment.^15^, ^16^ Nearly all studies in Latin America, Africa, and Asia confirm that women are marginally more likely to develop dementia and AD, particularly in very old age and increasing age is the most consistent risk factor for dementia worldwide.^16^ And higher levels of education were consistently associated with lower likelihood of developing cognitive impairment or dementia.^15^ It may because it is associated with healthy behaviors, better access to health care, higher‐paying occupations, and higher cognitive functioning.^17^, ^18^ Notably, the prevalence rate was slightly higher among those with more than 6 years of education than among those with 1–6 years of education in our data. We think the reason for this is that there are other confounding factors. Liuyang City is a developing city, with lower socioeconomic status, and some residents have the characteristics of poorer health and lower access to health care, especially in villages, which reduces the impact of the education level on cognition function. And perhaps it is related to the large span of years of education we set.

Regarding the risk factors associated with cognitive impairment, as expected, we found that lifestyle factors can indeed influence cognitive function.^19^ We found alcohol consumption was a protective factor for having a cognitive impairment and the effect persists in urban communities only. This may be due to higher income levels and greater frequency and variety of alcohol exposure in urban communities. We then quantified alcohol‐related measures, including frequency of drinking, daily alcohol intake, and years of drinking, but found no further association between drinking and cognitive impairment. This result is not surprising, as the relationship between alcohol consumption and cognitive impairment has been controversial. Studies have found that light‐to‐moderate alcohol intake may protect against dementia while excessive drinking may instead increase the risk.^20^ For instance, modest alcohol consumption (≤12.5 g/day) is associated with a reduced risk of dementia with 6 g/day of alcohol conferring a lower risk than other levels while excessive drinking (≥38 g/day) may instead elevate the risk.^20^ But the protective effect of alcohol consumption was also thought to be due to survivor bias.^12^ As for alcohol type, compared to beer and spirits, wine was more protective.^20^ Perhaps because the target population of our study had a relatively simple type of alcohol intake, more than 99% concentrated on spirits, we did not get further conclusions about alcohol intake. In addition, smoking is more likely to be a risk factor for cognitive impairment in epidemiological studies.^9^, ^21^ In prospective cohort studies, long‐term continuous smoking increases the risk of cognitive impairment in older age,^22^ and passive smoking exposure also increases the risk of cognitive impairment in older adults, especially non‐smokers.^8^ Smoking is also a risk factor for AD, and according to the prevalence of smokers worldwide, about 14% of AD cases may be attributed to this factor.^23^ But we found that increasing the number of cigarettes smoked per day was associated with a lower risk of cognitive impairment. As this is a cross‐sectional study, it is not possible to determine the causal relationship between smoking and cognitive impairment, and there is no overall consistency in our results, so it is not clear whether smoking has a protective effect on cognitive impairment. But our findings may suggest that smoking has a two‐way effect, perhaps related to the amount of smoking and the status of the target population.

In terms of comorbidities, it is found that older participants were more likely to present cognitive impairment that can be justified by the increase of chronic diseases^24^ or factors damaging the brain, such as head trauma.^25^ Cerebrovascular disease and Alzheimer's pathologies are key contributors to the overall burden of dementia.^26^, ^27^, ^28^ An analysis of 1079 individuals in the clinical‐pathologic Religious Orders Study and Memory and Aging Project found the isolated AD in only 9% versus 40% with AD plus an advanced vascular pathology (macroscopic infarcts, cerebral amyloid angiopathy, atherosclerosis, or arteriolosclerosis) and 44% with AD, vascular, and another neurodegenerative pathology.^29^ This is congruent with the positive association observed in this study between cognitive impairment and a history of cerebrovascular disease. And this association persisted among village participants, which may be due to the relative lack of medical resources and awareness, leading to an increased risk of cerebrovascular disease. A growing body of literature based on cross‐sectional and longitudinal data has shown that vision impairment is associated with cognitive decline among the older.^30^, ^31^, ^32^ Changes in multiple measures of vision such as visual acuity, sensitivity, and visual processing speed have been observed among the aging population, and these changes have been associated with cognitive impairments.^33^, ^34^ Our results corroborate these conclusions by showing that severe vision impairment is a risk factor for cognitive impairment in both villages and urban communities.

In conclusion, the results reconfirmed that the prevalence of cognitive impairment increases with the increase of age and decreases with the increase of years of education, and the prevalence of cognitive impairment is higher in villages than in urban communities. In addition, alcohol consumption and smoking emerged as protective factors, and patients with cerebrovascular disease or severe vision impairment were at higher risk. Our study updated cognitive impairment, MCI, and dementia prevalence data in a Han population in southern China, and we made some discoveries about risk factors, especially in lifestyle factors.

## AUTHOR CONTRIBUTIONS

Tianyan Xu was the first author of this work. Lu Shen and Yuzhang Bei had full access to all of the data in the study and took responsibility for the integrity of the data and the accuracy of the data analysis. Concept and design: Tianyan Xu, Yuzhang Bei, Lu Shen. Participant recruitment and data collection: Tianyan Xu, Guiwen Bu, Li Yuan, Lu Zhou, Qijie Yang, Yuan Zhu, Sizhe Zhang, Qianqian Liu, Ziyu Ouyang, Xuan Yang, Bin Jiao. Cognitive function assessment: Tianyan Xu, Qijie Yang, Yuan Zhu, Sizhe Zhang, Xuan Yang, Bin Jiao. Drafting of the manuscript: Tianyan Xu. Critical revision of the manuscript: Tianyan Xu, Bin Jiao, Beisha Tang, Yuzhang Bei, Lu Shen. Statistical analysis: Tianyan Xu. Supervision: Yuzhang Bei, Lu Shen.

## FUNDING INFORMATION

This study was supported by the National Key R&D Program of China (No. 2020YFC2008500), STI2030‐Major Projects (No. 2021ZD0201803), and the National Natural Science Foundation of China (Nos. U22A20300, 81971029, 82071216, 81901171).

## CONFLICT OF INTEREST STATEMENT

The authors declare that they have no competing interests.

## Data Availability

The data that supports the findings of this study are available in the materials of this article. Other information is available from the corresponding author on reasonable request.
